# Help-Seeking Patterns Among Socially Isolated Patients in Primary Care

**DOI:** 10.1007/s11606-025-10130-7

**Published:** 2026-01-08

**Authors:** Minal R. Patel, Cindy W. Leung, Rhea Saksena, Wei Hao

**Affiliations:** 1 Department of Health Behavior & Health Equity, University of Michigan School of Public Health, Ann Arbor, MI USA; 2 Department of Nutrition, Harvard T.H. Chan School of Public Health, Boston, USA; 3 Department of Biostatistics, University of Michigan School of Public Health, Ann Arbor, MI USA

**Keywords:** social isolation, help-seeking, social determinants of health, primary care

## INTRODUCTION

Social isolation significantly impacts physical and mental health outcomes,^1,2^ prompting healthcare systems to implement social needs screening programs. However, over half of patients screening positive for unmet social needs decline assistance or fail to follow up with resources.^3,4^ Understanding help-seeking patterns among socially isolated patients is crucial for optimizing resource allocation and care delivery, yet limited research has examined the intersection between social isolation screening results and patients' willingness to accept assistance. This study aimed to examine the prevalence of social isolation and assistance-seeking patterns, and identify demographic and clinical characteristics associated with requesting assistance among socially isolated patients.

## METHODS

We conducted a retrospective analysis of electronic health records from a systematic social needs screening program implemented across a large integrated healthcare system from August 2017 to December 2024. This study was considered exempt by the University of Michigan institutional review board, and informed consent was not needed because the data were anonymous. The study population included all primary care patients aged ≥ 18 years who completed social needs screening during routine visits.

Social isolation was assessed using a validated single-item screener:^5^ “Do you feel isolated from others?” administered with three timeframes: past 12 months, currently, and past 4 weeks. Response options were binary (yes/no). Patients were classified as socially isolated if they responded “yes” to any timeframe.

The primary outcome was interest in assistance, assessed with two questions: (1) “Do you want to be contacted by the Guest Assistance Program for help on any of your responses?” and (2) “Do you want to get connected with resources for isolation?” (y/n).

Demographic and clinical characteristics, and co-occurring social needs (categorized as 0, 1–2, or ≥ 3) were also examined. Among socially isolated patients, adjusted multivariable logistic regression identified predictors of requesting assistance, calculating adjusted odds ratios (AOR) with 95% confidence intervals.

## RESULTS

Among 110,995 patients, 25,067 (22.6%) screened positive for social isolation. Of patients with complete assistance data (*n* = 60,034), only 1,860 of 21,256 socially isolated patients (8.8%) requested assistance.

Socially isolated patients were more likely to be female (63.9% vs. 56.3%), single (37.7% vs. 25.2%), or divorced/widowed (11.0% vs. 7.3%). Social isolation rates were higher among Non-Hispanic Black patients (8.7% vs. 6.6%) and lower among Asian patients (5.9% vs. 9.2%). Isolated patients had higher rates of Medicaid coverage (10.9% vs. 5.8%), severe obesity (BMI ≥ 35: 20.6% vs. 14.8%), and co-occurring social needs (Table 1).

**Table 1:** Demographic and Health Characteristics of Total Sample and Those Reporting and not Reporting Social Isolation

		Social isolation N (%)		
	Total Patients (*N* = 110995) *N* (%)	Yes (*N* = 25067) *N* (%)	No (*N *= 85928) *N* (%)	*P*-value
Age, mean (SD), y	53.1 (17.6)	50.6 (18.6)	53.8 (17.3)	< 0.0001
Sex, N (%)				< 0.0001
Female	64,426 (58.0)	16,010 (63.9)	48,416 (56.3)	
Male	46,569 (42.0)	9057 (36.1)	37,512 (43.7)	
Race and ethnicity, N (%)				< 0.0001
Non-Hispanic White	81,259 (79.7)	81,259 (79.8)	62,884 (79.6)	
Non-Hispanic Black	7229 (7.1)	7229 (8.7)	5237 (6.6)	
Hispanic	3251 (3.2)	865 (3.8)	2386 (3.0)	
Asian	8651 (8.5)	1356 (5.9)	7295 (9.2)	
Other	1632 (1.6)	430 (1.9)	1202 (1.5)	
Marital status				< 0.0001
Married or significant other	60,430 (63.9)	10,752 (51.3)	49,678 (67.5)	
Single	26,455 (28.0)	7887 (37.7)	18,568 (25.2)	
Divorced or widowed	7697 (8.1)	2311 (11.0)	5386 (7.3)	
Insurance type, N (%)				< 0.0001
Private	79,362 (71.5)	17,134 (68.3)	62,228 (72.4)	
Medicare	19,177 (17.3)	4227 (16.9)	14,950 (17.4)	
Medicaid	7686 (6.9)	2733 (10.9)	4953 (5.8)	
Uninsured	3426 (3.1)	698 (2.8)	2728 (3.2)	
Other	1344 (1.2)	275 (1.1)	1069 (1.2)	
BMI				< 0.0001
BMI < 25	33,429 (30.7)	7285 (29.6)	26,144 (31.0)	
25 ≤ BMI < 30	36,046 (33.1)	7266 (29.6)	28,780 (34.1)	
30 ≤ BMI < 35	21,999 (20.2)	4979 (20.3)	17,020 (20.2)	
BMI $\ge$ 35	17,538 (16.1)	5059 (20.6)	12,479 (14.8)	
Co-Occurring Social Needs				< 0.0001
0	95,185 (85.8)	16,764 (66.9)	78,421 (91.3)	
1–2	13,482 (12.2)	6588 (26.3)	6894 (8.0)	
≥ 3	2328 (2.1)	1715 (6.8)	613 (0.7)	
Other material strain, N (%)	12,426 (11.2)	6597 (26.3)	5829 (6.8)	< 0.0001
Caregiving/interpersonal problems, N (%)	5607 (5.1)	3356 (13.4)	2251 (2.6)	< 0.0001

Among socially isolated patients, several groups were more likely to request assistance: older adults (ages 65–74: AOR 1.36, 95% CI 1.09–1.70; ≥ 75: AOR 1.75, 95% CI 1.36–2.25), unmarried patients (single: AOR 1.58, 95% CI 1.38–1.81; divorced/widowed: AOR 1.48, 95% CI 1.23–1.77), non-white patients (Non-Hispanic Black: AOR 1.75, 95% CI 1.49–2.07; Hispanic: AOR 1.47, 95% CI 1.11–1.94; Asian: AOR 1.84, 95% CI 1.46–2.30), and publicly insured patients (Medicare: AOR 1.42, 95% CI 1.19–1.69; Medicaid: AOR 1.56, 95% CI 1.32–1.83) (Table 2).

**Table 2:** Predictors of Requesting Assistance Among Socially Isolated Patients (*N* = 21,256)

	AOR (95% CI)	*P*-value
Age		
18–34	Ref	
35–44	0.94 (0.78, 1.15)	0.55
45–54	1.14 (0.94, 1.38)	0.19
55–64	1.19 (0.98, 1.45)	0.08
65–74	1.36 (1.09, 1.70)	0.006*
≥ 75	1.75 (1.36, 2.25)	< 0.0001*
Sex		
Female	Ref	
Male	1.06 (0.94, 1.20)	0.35
Marital status		
Married or significant other	Ref	
Single	1.58 (1.38, 1.81)	< 0.0001*
Divorced or widowed	1.48 (1.23, 1.77)	< 0.0001*
Race and ethnicity		
Non-Hispanic White	Ref	
Non-Hispanic Black	1.75 (1.49, 2.07)	< 0.0001
Hispanic	1.47 (1.11, 1.94)	0.008*
Asian	1.84 (1.46, 2.30)	< 0.0001*
Other	1.29 (0.87, 1.91)	0.21
Insurance type		
Private	Ref	
Medicare	1.42 (1.19, 1.69)	< 0.0001*
Medicaid	1.56 (1.32, 1.83)	< 0.0001*
Uninsured	1.09 (0.77, 1.53)	0.63
Other	1.42 (0.84, 2.38)	0.19
Co-Occurring Social Needs		
0	Ref	
1–2	3.18 (2.78, 3.62)	< 0.0001*
≥ 3	9.10 (7.67, 10.80)	< 0.0001*

The strongest predictor was co-occurring social needs. Patients with 1–2 needs (AOR 3.18, 95% CI 2.78–3.62) and ≥ 3 needs (AOR 9.10, 95% CI 7.67–10.80) were more likely to request assistance than those with no needs.

## CONCLUSION

While nearly one in four patients experience social isolation, only 10% request assistance. The most critical finding is that patients with multiple co-occurring social needs were nine times more likely to seek help, suggesting that isolation alone may be perceived differently than isolation combined with material hardships.

This pattern means that socially isolated patients without other material needs are most likely to remain undetected. Contrary to expectations,^6^ traditionally vulnerable populations were more likely to seek help when isolated, likely because they experience isolation alongside other pressing needs.

Healthcare systems should implement proactive strategies to capture patients who screen positive for isolation but lack other co-occurring social needs. Current passive screening approaches may systematically miss these patients who appear demographically advantaged and lack obvious material hardships.

Study limitations include the retrospective design limiting causal inference, potential unmeasured confounding, and limited generalizability from a single healthcare system. These findings underscore the need for healthcare systems to redesign screening approaches to identify and engage socially isolated patients who may not self-advocate, addressing a significant challenge affecting nearly one-quarter of patients.

## Data Availability

The data for these study are from the EHR, and therefore cannot be shared.
